# From the 3rd to the 7th year after straw return, different straw-returning practices drive shifts in soil fungal community composition, functional differentiation, and the reconfiguration of community assembly processes

**DOI:** 10.3389/fmicb.2026.1808010

**Published:** 2026-04-21

**Authors:** Rui-Zhi Liu, Xiao-Ya Zhao, Xiao-Yu Zhao, Wen-Shan Zhao, Shu-Ping Hu, Rui-Ping Li, Xiao-Fang Yu, Ju-Lin Gao, Qinggeer Borjigin

**Affiliations:** 1 Inner Mongolia Agricultural University, Hohhot, China; 2 Key Laboratory of Crop Cultivation and Genetic Improvement of Inner Mongolia Autonomous Region, Hohhot, China; 3 Inner Mongolia Autonomous Region Engineering Research Centre of Microorganisms for in Situ Corn Straw Return, Hohhot, China; 4 Institute of Maize Research, Inner Mongolia Academy of Agricultural & Animal Husbandry Sciences, Hohhot, China; 5 Nongye Bureau of Jingyang District, Deyang, Sichuan, China; 6 Vocational and Technical College, Inner Mongolia Agricultural University, Baotou, China

**Keywords:** community assembly mechanism, community succession, soil fungi, straw return, tillage practice

## Abstract

**Introduction:**

In long-term straw-returning systems, a year-scale understanding of how contrasting tillage practices shape soil fungal succession and community assembly remains limited.

**Methods:**

Based on a long-term field experiment, we investigated soil fungal communities using ITS sequencing during years 3–7 after straw return (2020–2024) under farmer shallow rotary tillage (CK) and three treatments: deep ploughing return (DPR), subsoiling straw return (SSR), and no-tillage mulching return (NTR). Fungal diversity, community composition, functional guilds, and assembly pathways were evaluated by integrating functional guild assignment, co-occurrence network analysis, and null-model metrics (βNTI) with a neutral community model.

**Results:**

Fungal *α*-diversity showed a pronounced mid-term increase (years 4–5; +18–40% in Shannon index) and stabilized thereafter (variation <15%), indicating a transition toward community equilibrium. Community composition exhibited directional turnover, with Ascomycota decreasing (~31–44%) and Basidiomycota increasing (up to ~226–228%). By year 7, clear treatment-specific differences emerged: Ascomycota was higher in DPR than in SSR (+62.96%), whereas Blastocladiomycota increased markedly in NTR (4.49–31.40-fold). At the genus level, DPR enriched *Trichosporiella* (up to 29.74-fold higher than NTR), while *Solicoccozyma* was more abundant in SSR and NTR (2.94–3.00-fold higher than DPR). Functionally, DPR increased symbiotic guilds (+90.81%), whereas SSR and NTR showed higher pathogen-associated guilds (e.g., SSR 1.63-fold higher than DPR). Network analysis revealed that NTR formed the largest network but with stronger pathogen-associated signals, whereas DPR showed higher cooperativity (93.61% positive edges) and stability. Assembly analyses indicated overall stochastic dominance, with increased deterministic processes in NTR in year 5 (βNTI > 2). The neutral model showed moderate fit (R^2^ = 0.5132), with greater deviation under NTR. Soil microbial biomass, enzyme activities, soil organic matter, and moisture were key drivers of community shifts.

**Discussion:**

These results demonstrate that contrasting straw-returning practices regulate fungal succession through compositional turnover, functional differentiation, and assembly reconfiguration, providing insights for optimizing straw-return management and promoting sustainable cropland systems.

## 1 Introduction

With continued global population growth and rising living standards, the demand for crops has increased steadily. In China, grain production increased by 3.9-fold from 1961 to 2011 (Cui et al., 2014), and is projected to further increase by 30–50% by 2030 due to population growth, rising incomes, and dietary changes (Battaglia et al., 2021; Li Y. et al., 2018). This sustained increase in crop production has led to a corresponding rise in crop straw generation. In recent years, global annual crop straw production has reached approximately 4.0 billion tons, with China accounting for nearly one third (Li H. et al., 2018; Chen et al., 2020). The large volume of crop residues presents both challenges and opportunities for agricultural systems. Efficient utilization of crop straw resources not only improves resource-use efficiency but also contributes to emission reduction, carbon sequestration, and the maintenance of soil ecological functions.

Soil microbial ecology studies show that microbial community diversity, composition, and ecological functions exhibit highly dynamic respond dynamically to agricultural management practices (Carrasco-Espinosa et al., 2022). Among these microorganisms, soil fungi play essential roles in agroecosystems by actively participating in organic matter decomposition, nutrient cycling, plant symbioses, and pathogen suppression, thereby contributing to soil health and functional stability (Guang et al., 2025; Wang et al., 2024a). Particularly in semi-arid regions, fungi are more sensitive to environmental stressors than bacteria and are therefore considered key indicators of soil ecosystem health (Qiang et al., 2024).

Previous studies have demonstrated that straw-returning practices can significantly increase microbial *α*-diversity by enhancing soil organic carbon inputs and improving habitat heterogeneity, while also reshaping *β*-diversity through changes in environmental filtering and resource availability (Ye et al., 2024). At the compositional level, straw incorporation has been reported to increase the relative abundance of dominant fungal phyla such as Ascomycota and Basidiomycota, while promoting specific functional groups involved in lignocellulose degradation (e.g., members of *Sordariomycetes*) (Zong et al., 2023). In addition, straw mulching or reduced tillage practices can selectively enrich saprotrophic and symbiotic taxa, while in some cases increasing the abundance of potential pathogenic genera such as Fusarium under certain management regimes (Schmidt et al., 2019).

In semi-arid agroecosystems, limited precipitation and strong evaporation can reduce organic matter inputs while accelerating its mineralization, and pronounced temperature fluctuations may destabilize soil structure and microbial activity, thereby contributing to declines in soil organic matter and soil fertility. Previous studies have systematically examined how different straw-returning practices affect soil physicochemical properties [e.g., pH, soil organic matter (SOM), and bulk density (BD)] and have further explored their impacts on microbial community diversity and composition (Zhang et al., 2025). However, how different straw-returning practices regulate fungal community succession and assembly processes over multiple years remains poorly understood, particularly under semi-arid conditions.

With the advancement of sustainable agricultural management, straw return has been widely adopted to enhance nutrient cycling and improve soil quality in croplands. Different straw-returning practices (i.e., deep ploughing return, DPR; subsoiling straw return, SSR; and no-tillage mulching return, NTR) can substantially alter the soil physicochemical environment, microbial substrate availability, and microsite structure, thereby markedly shaping fungal diversity, community structure, and assembly mechanisms (Liu et al., 2025). Deep ploughing return (DPR) generally enhances fungal *α*-diversity by improving soil aeration and promoting organic matter mixing, whereas no-tillage mulching return (NTR) provides a more stable habitat that favors symbiotic fungi and niche differentiation (Kang et al., 2025; Schlatter et al., 2018).

Microbial *β*-diversity is jointly shaped by interannual variation and straw-returning practices, indicating potential ecological memory effects under long-term management (Kang et al., 2025; Xu et al., 2025). Studies indicate that community assembly mechanisms are sensitive to management practices: straw-returning practices can shift the relative importance of stochastic processes (e.g., neutral dispersal) and deterministic processes (e.g., environmental filtering), thereby reshaping fungal assembly trajectories (Li et al., 2025; Lian et al., 2025). Under straw-returning conditions, fungal community assembly is often dominated by stochastic processes, whereas NTR may enhance deterministic selection during specific period (Cui et al., 2025). Additionally, co-occurrence network analyses indicate that straw-returning practices can markedly alter fungal network architecture and ecological stability, with no-tillage straw mulching increasing node connectivity and network complexity (Su et al., 2025; Ya et al., 2023). Shallow tillage with chopped straw incorporation has been shown to strengthen positive (cooperative) associations within the co-occurrence network (Ding et al., 2025).

Therefore, this study was conducted in a typical continuous maize cropping system in Inner Mongolia using a long-term field experiment established in 2018. We systematically compared, over 2020–2024, three straw-returning practices—deep ploughing with chopped straw incorporation (DPR), subsoiling with straw mixing (SSR), and no-tillage straw mulching (NTR)—with a straw removal treatment under farmer shallow rotary tillage (CK, control), to quantify their effects and spatiotemporal dynamics on soil fungal diversity, community composition, assembly processes, ecological functions, and network topological features. Specifically, we addressed three questions: (1) How do different straw-returning practices influence fungal *α*- and *β*-diversity and dominant taxa? (2) Do straw-returning practices significantly alter fungal assembly processes and ecological network architecture? (3) How do key drivers, including soil physicochemical properties, nutrient status, and enzyme activities, regulate fungal functional attributes and network stability? To answer these questions, we combined high-throughput sequencing with functional guild inference and applied a suite of analytical frameworks, including the neutral community model (Sloan model), the β nearest taxon index (βNTI), iCAMP, co-occurrence network analysis, and partial least squares path modeling (PLS-PM), to elucidate the ecological processes governing soil fungal responses to contrasting straw-returning practices.

## 2 Materials and methods

### 2.1 Experimental site

This study is based on the pre-constructed straw return trial platform of the group (the straw returning platform test site was established in 2018) conducted in 2020–2024 and was carried out in the China Chilechuan Modern Agricultural Expo Park (Beizhitu Village, Goumen Town, Tumet Right Banner, Baotou City, Inner Mongolia, latitude 40°28′28″N, longitude 110°29′5″E), where perennial straw return to the experimental field has been implemented starting in 2018. This area has a semi-arid mesothermal temperate continental monsoon climate, with an average annual temperature of 6–8 °C, 400 mm of annual precipitation, a frost-free period of 140 days, an elevation of 1,015 m, 2,806 h of annual sunshine, and an annual active cumulative temperature ranging from 3,000–3,500 °C. The site is used for continuous maize cultivation. In the absence of tillage, the soil texture is sandy loam, and the soil fertility is characterized by an organic matter content of 22.04 g/kg, an alkali-hydrolysable nitrogen content of 57.82 mg/kg, an available phosphorus content of 3.57 mg/kg, and an available potassium content of 84.97 mg/kg. The soil nutrient data collected before sowing and tillage (0–45 cm soil layer) are shown in Supplementary Table S1, and the main meteorological data collected during the test period are shown in Supplementary Figure S1.

### 2.2 Experimental design

The experiment used a one-factor experimental design, where the plowing method was applied in the central zone. Farmers’ shallow rotation (CK): refers to the sowing method used by local farmers. During the fall land preparation phase, the straw is crushed and baled, then removed from the field. In spring, after the stubble is processed, conventional shallow rotation sowing is carried out. Three treatments were established for comparison, straw incorporated with deep tillage (DPR): in autumn, straw is shredded and plowed into the soil to a depth of 30–40 cm. In spring, sowing is done using a conventional planter. Straw incorporated with subsoiling (SSR): in autumn, straw is shredded and mixed with the soil after subsoiling to a depth of 35–40 cm. In spring, sowing is done using a conventional planter. No-tillage mulching straw return (NTR): in autumn, straw is shredded and left on the soil surface as mulch. In spring, sowing is done using a no-till planter. All straw return treatments included full corn straw return at 135–150 kg ha − 1. The maize variety planted was Xianyu 696 at a planting density of 825 plants/ha. Ammonium dihydrogen phosphate (N 18%, P 46%) was applied at a rate of 375 kg ha − 1, and potassium sulfate (K 22%) was applied at a rate of 150 kg ha − 1. Urea (N 46%) was applied utilized as a supplementary fertilizer with application rates of 30% at V6 (sixth leaf), 60% at V12 (twelfth leaf), and 10% at R2 (blister), resulting in a total nitrogen application of 345 kg/ha. Drip irrigation was performed four times during the growing season: at V6, V12, R1 (silking), and R2. Each irrigation event covered 750 m3/ha. All remaining management practices followed standard procedures commonly used in large-scale agricultural production.

The experiment was conducted annually during the maize pre-sowing period from 2020 to 2024. Soil samples were collected each May, totaling four sampling events. In each experimental plot, soil was collected from the 0–45 cm layer using an imported auger in an “S”-shaped sampling pattern. A total of 12 subsamples were taken per plot, thoroughly mixed, and passed through a 2 mm mesh sieve to remove plant debris, root fragments, and other impurities. The homogenized soil was then subjected to the quartering method to obtain a representative sample, sealed in sterile bags, and immediately transported to the laboratory. Each composite sample was divided into three parts: the first portion was air-dried for determining soil physicochemical properties and measuring the activity of alkaline phosphatase (ALP) (Tan et al., 2017; Wang Z. Q. et al., 2017) and catalase, the latter being assessed by back-titration of residual H₂O₂ with KMnO₄ (Johnson and Temple, 1964). The second portion was stored at 4 °C for analysis of glutamine synthetase (Glu) activity (Haghighat, 2005), the third portion was stored at −80 °C for DNA extraction and high-throughput sequencing.

### 2.3 Soil properties analysis

Soil Moisture (SM,%) was measured using the JL-01 multi-point soil temperature and humidity recorder (JL-01, Jingyi Electronic Company). Soil Bulk Density (g/cm^3^, BD) was determined by the core method (VanRemortel and Shields, 1993). Soil Alkaline Nitrogen (AN) was measured using the alkaline hydrolysis diffusion method (Bremner, 1960). Available Phosphorus (AP) was determined with the Smartchem140 automated chemical analyzer (SMARTCHEM450, AMS, France) (Olsen and Sommers, 1982). Available Potassium (AK) was measured using a flame photometer (M410, SHERWOOD SCIENTIFIC, United States) (Page et al., 1982). Soil organic matter (SOM) was determined by the potassium dichromate metho (Walkley and Black, 1934). Alkaline Phosphatase (ALP) was determined by the phenylphosphate colorimetric method (Liu et al., 2020). Hydrogen Peroxide Decomposing Enzyme (H₂O₂) was measured by the potassium permanganate titration method (Borràs-Brull et al., 2021). Glutamine synthetase (GS) was determined using visible spectrophotometry (Haghighat, 2005).

### 2.4 Sequencing and bioinformatics analysis

#### 2.4.1 DNA extraction, PCR amplification, and sequencing

Soil fungal DNA was extracted from soil samples using an E.Z.N.A.^®^ Soil DNA Kit (Omega Biotek, Norcross, GA, United States) according to the manufacturer’s instructions. The extracted DNA was quantified using a 1.0% (w/v) agarose gel and a NanoDrop 2000 spectrophotometer (Thermo Scientific, Wilmington, United States). The fungal ITS1 region was amplified using the primer pair ITS1F (5′-CTTGGTCATTTAGAGGAAGTAA-3′) and ITS2R (5′-GCTGCGTTCTTCATCGATGC-3′) with an ABI GeneAmp^®^ 9,700 PCR thermocycler (ABI, CA, United States) (Zhang Y. et al., 2016). PCR amplification was performed in a 20 μL reaction mixture containing 4 μL of 5 × FastPfu buffer, 2 μL of 2.5 mM dNTPs, 0.8 μL of each primer (5 μM), 0.4 μL of FastPfu DNA polymerase, 0.2 μL of BSA, 10 ng of template DNA, and ddH₂O to volume. The PCR program consisted of an initial denaturation at 95 °C for 3 min, followed by 35 cycles of 95 °C for 30 s, 55 °C for 30 s, and 72 °C for 45 s, with a final extension at 72 °C for 10 min. PCR products were extracted from a 2% agarose gel, purified using the AxyPrep DNA Gel Extraction Kit (Axygen Biosciences, Union City, CA, United States), and quantified with a Quantus^™^ Fluorometer (Promega, United States) (Zhang et al., 2022). Purified amplicons were pooled in equimolar concentrations and subjected to paired-end sequencing on the Illumina MiSeq platform (Illumina, San Diego, USA) by Majorbio Bio-Pharm Technology Co., Ltd. (Shanghai, China). The raw sequencing data have been deposited in the NCBI database under accession number PRJNA140523.

#### 2.4.2 Sequencing data processing

Raw fastq files were demultiplexed, quality-filtered using Trimmomatic (Aachen, Germany), and merged using FLASH (San Francisco, USA). Operational taxonomic units (OTUs) were clustered at a 97% similarity cutoff using UPARSE (version 7.1, https://drive5.com/uparse/), and chimeric sequences were identified and removed using UCHIME. The taxonomy of each ITS sequence was assigned against the UNITE fungal ITS database using the RDP Classifier^1^ with a confidence threshold of 70%.

### 2.5 Assembly processes analysis

To classify fungal community assembly into underlying deterministic and stochastic processes, the *β*-nearest taxon index (βNTI) was calculated using a null model generated with 999 randomizations based on the observed data (OTU table and phylogenetic tree) (Jiao et al., 2022; Shi et al., 2023). βNTI < −2 and βNTI > 2 indicated homogeneous and heterogeneous selection (deterministic processes), respectively. |βNTI| < 2 indicated that bacterial community assembly was the dominance of stochastic processes. To further quantify the relative contributions of ecological processes, the iCAMP framework was applied as a phylogenetic bin-based null model approach, enabling a finer partitioning of deterministic and stochastic assembly processes (Ning et al., 2020).

### 2.6 Co-occurrence network analysis and ZiPi analysis

A fungal OTU-level co-occurrence network was constructed using Spearman’s rank correlation (Shi et al., 2023). Only OTUs with relative abundance > 0.1% were used in the analyses. Statistical correlations were identified when Spearman’s r > 0.7 or < −0.7 and *p* < 0.05 and were then incorporated into the co-occurrence network construction. The node and edge numbers, average clustering coefficient, average degree and graph density were used to evaluate network complexity in this study. Positive and negative correlations between nodes represented cooperative and competitive behaviors (Wang C. et al., 2024).

For the ZiPi analysis, the igraph package and the NetCoMi package (version 1.0.4) in R were used (Olesen, et al., 2007). Based on the Zi value (within-module connectivity) and Pi value (among-module connectivity) of each node, the network nodes were classified into four categories: (1) Module Hubs (Zi > 2.5, Pi ≤ 0.62), which occupy important positions within modules but have weak associations with other modules, (2) Connectors (Zi > 2.5, Pi > 0.62), which play a significant role in the functional connectivity of the entire network, (3) Within-module Non-hubs (Zi ≤ 2.5, Pi ≤ 0.62), and (4) Module Outside Connectors (Zi ≤ 2.5, Pi > 0.62; Guimerà and Nunes Amaral, 2005).

### 2.7 Statistical analysis

Statistical analyses were conducted using SPSS 22.0 (IBM SPSS, Chicago, IL, United States) and R software (version 4.3.2). Descriptive statistics were determined for soil properties, including mean, maximum, and minimum values and coefficient of variation (CV) (Zhang H. et al., 2016) Alpha-diversity indices, including the nonparametric richness estimator ACE and the Shannon diversity index, were calculated from OTU data using Mothur (v1.30.2). Two-way ANOVA was used to estimate soil chronosequence and landuse effects on microbial alpha diversity (Wang et al., 2023). Non-metric multidimensional scaling (NMDS) plots and analysis of similarity (ANOSIM) test with 999 permutations based on Bray Curtis distancemetric in R with the Vegan package were used to visualize and assess the effects of soil chronosequence and land use on soil microbial community composition (Huang et al., 2019). Soil fungi were analyzed using FUN Guild. To investigate the association between soil microbial community composition and environmental factors, redundancy analysis (RDA) was employed. A Mantel test with a Monte Carlo simulation of 999 randomizations was performed. Partial least squares path model (PLS-PM) in the plspm package was used to explore the relationships among reclamation duration, soil properties, microbial diversity, and network complexity. This method is particularly effective for elucidating causal relationships between observed and latent variables (Tang et al., 2023). The goodness of fit (GOF) index indicated that our models had good overall predictive performance.

## 3 Results

### 3.1 Soil fungal α-diversity

**Figure 1:**
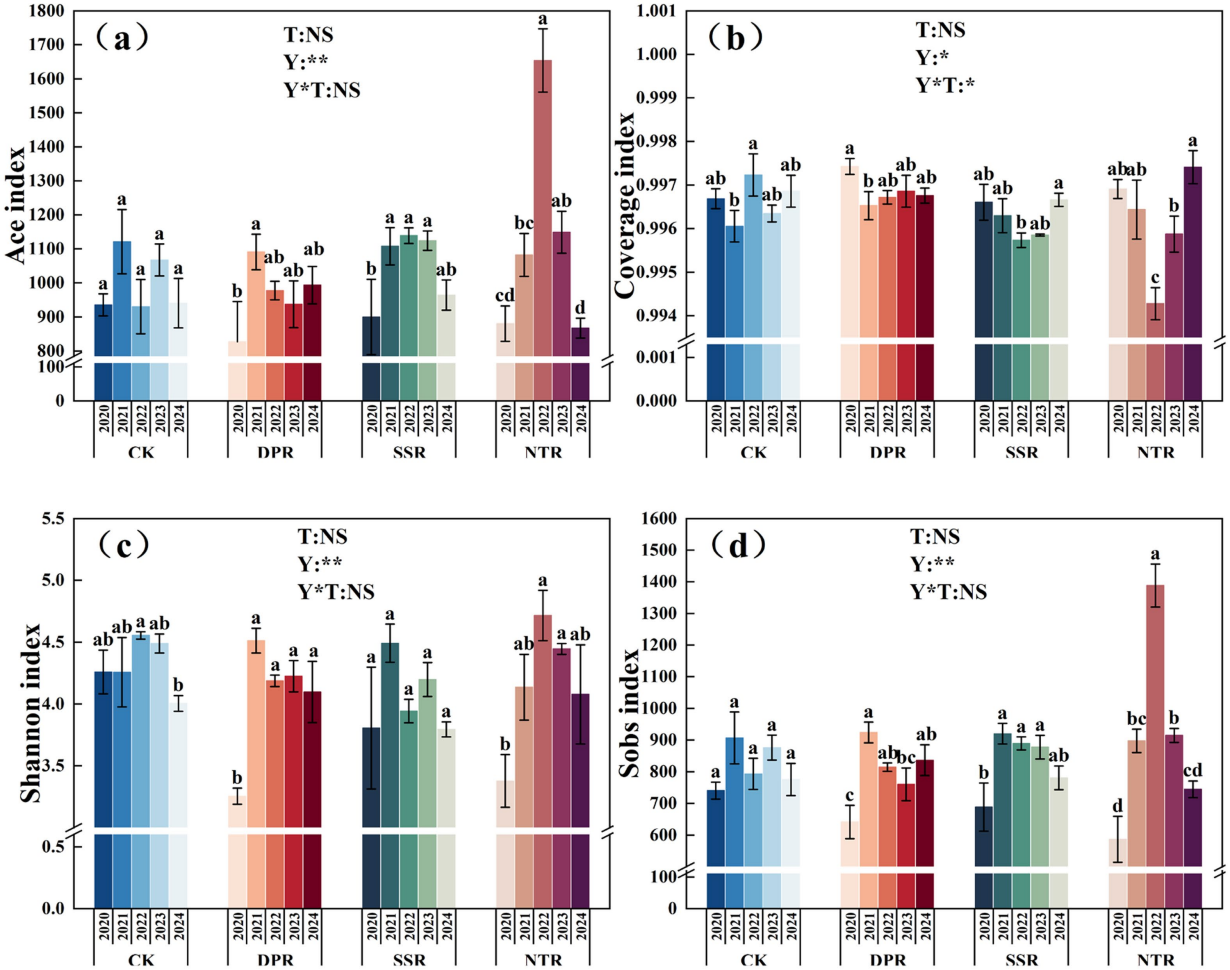
Changes in ace, coverage, Shannon, and sobs indices of soil fungal communities under different straw-returning practices from 2020 to 2024 along the soil time series. For a given treatment, different lowercase letters indicate significant differences among years (*p* < 0.05), whereas within a given year, different uppercase letters indicate significant differences among treatments (*p* < 0.05). Error bars represent standard deviation (SD) based on three replicates. For a given treatment, different lowercase letters indicate significant differences among years (*p* < 0.05), whereas within a given year, different uppercase letters indicate significant differences among treatments (*p* < 0.05).

In 2024, no significant differences were detected in fungal *α*-diversity indices among tillage practices (*p* > 0.05). Interannual analysis showed that all α-diversity metrics varied significantly over the study period (*p* < 0.05). During 2020–2022, the Ace and Sobs indices under the straw-return treatments increased by 25–40% relative to 2020, and the Shannon index increased by 18–40%; however, these indices declined by 8–15% in 2023–2024. Compared with CK, DPR, SSR, and NTR exhibited significantly higher α-diversity levels in 2021–2022 (*p* < 0.05), whereas the differences among treatments were no longer significant by 2024 (*p* > 0.05). Coverage values exceeded 0.994 for all treatments, with fluctuations of <0.3%. Meanwhile, straw-returning practices significantly improved soil physicochemical properties and enzyme activities compared with CK (Supplementary Table S2). Specifically, soil organic matter (SOM), available nitrogen (AN), available potassium (AK), and soil moisture increased by 64.81, 35.12, 58.67, and 42.36%, respectively, while bulk density decreased by 9.38%. Enzyme activities were also markedly enhanced, with GS and ALP increasing by 128.75 and 691.31%, respectively, particularly under DPR and SSR treatments (Supplementary Table S3). Overall, under long-term straw return, soil fungal α-diversity showed pronounced stage-dependent variation and tended to stabilize in the later years (Figure 1).

### 3.2 Soil fungal β-diversity

**Figure 2:**
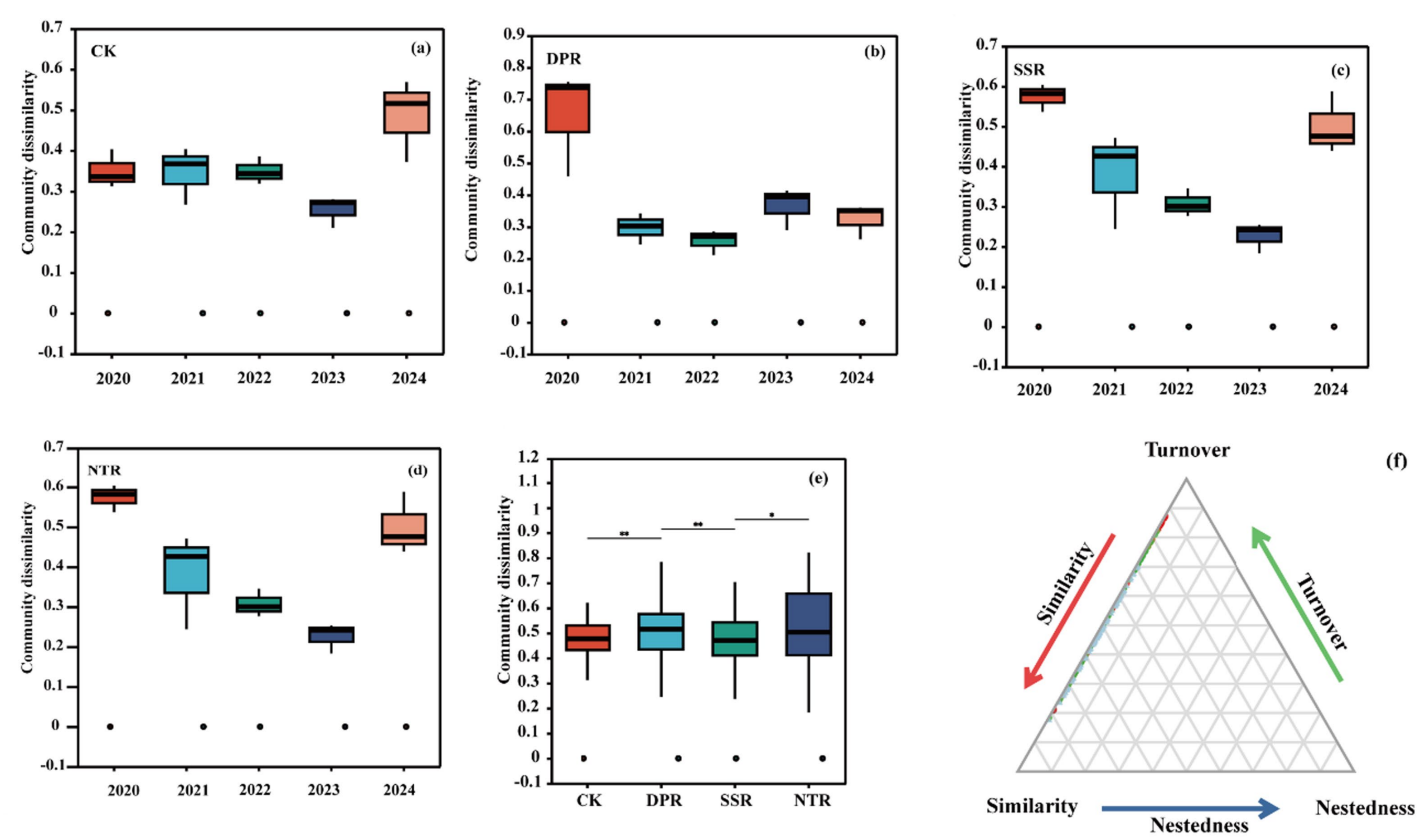
The NMDS ranking and similarity analysis (ANOSIM and ADONIS) tests show the differences in the true community structure of different straw application methods during the period from 2020 to 2024. Error bars represent standard deviation (SD) based on three replicates. For a given treatment, different lowercase letters indicate significant differences among years (*p* < 0.05), whereas within a given year, different uppercase letters indicate significant differences among treatments (*p* < 0.05).

PERMANOVA indicated that Year explained a larger proportion of the variation than Treatment (R^2^ = 0.224 vs. 0.168, *p* = 0.001). Together, Year and Treatment accounted for 39.2% of the total variation, with 60.8% remaining unexplained (residuals). In the β-diversity partitioning, the nestedness component was zero across all treatments. In the seventh year after straw return (2024), β-diversity did not differ significantly among treatments. When compared across years within each treatment, β-diversity also showed no significant interannual differences; however, annual means exhibited the following patterns: NTR was highest in 2020 and lowest in 2023, then rebounded to 0.501 in 2024; SSR declined from 0.401 in 2020 to 0.241 in 2024; DPR was highest in 2020 and lowest in 2022, increased to 0.366 in 2023, and was 0.324 in 2024; CK remained similar during 2020–2022, decreased to 0.254 in 2023, and increased to 0.486 in 2024. Based on annual means, the overall ordering was DPR > NTR > CK > SSR (see Figure 2; Table 1).

**Table 1:** PERMANOVA results for the effects of tillage treatment (treatment) and year (year) on soil fungal community β-diversity (2020–2024).

Characteristics	Df	SumsOfSqs	MeanSqs	F_Model	R^2^	Pr(>F)
Treatment	3	1.48533	0.49511	4.79738	0.16815	0.001
Year	4	1.98145	0.49536	4.79984	0.22431	0.001
Residuals	52	5.3666	0.1032	0	0.60754	0
Total	59	8.83337	0	0	1	0

### 3.3 Changes in soil fungal community composition under different straw-returning practices

**Figure 3:**
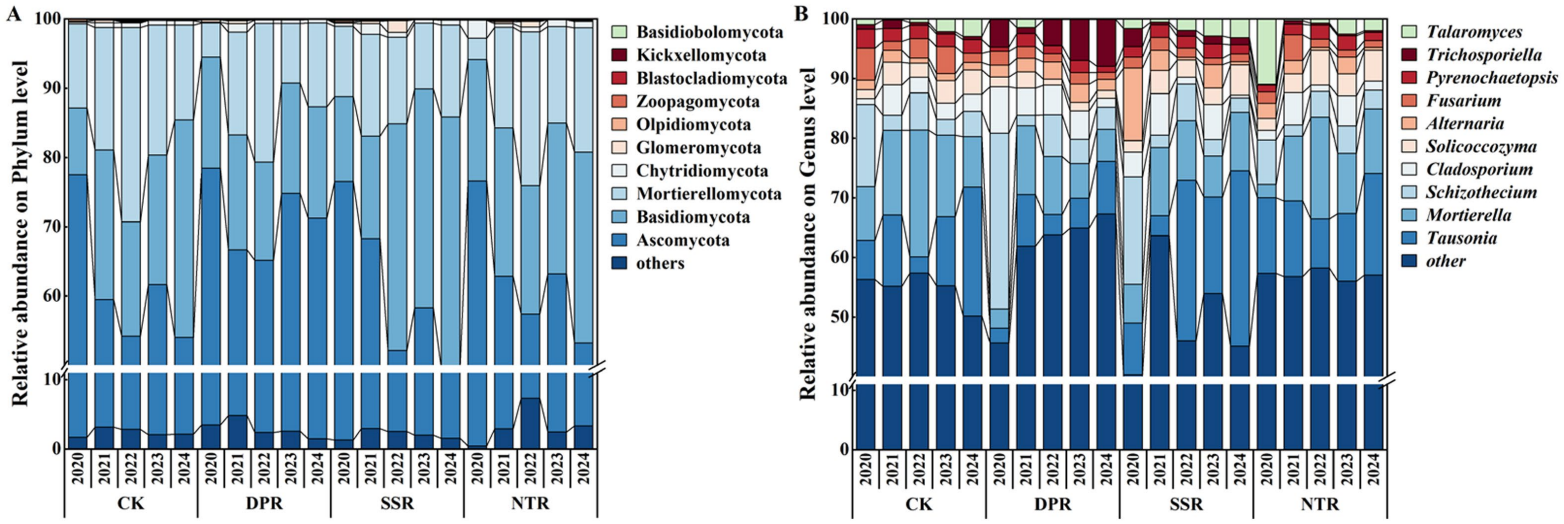
Relative abundances of the top 10 fungal phyla **(A)** and the top 10 fungal genera **(B)** under different straw-returning practices from 2020 to 2024.

Both straw-returning practice (T) and year (Y) significantly affected fungal community relative abundance. The relative abundances of Ascomycota, Basidiomycota, Mortierellomycota, and Glomeromycota, as well as the genera *Fusarium* and *Trichosporiella*, differed significantly among treatments and years (Figure 3A, *p* < 0.05). In the among-treatment comparison for 2024, Ascomycota under DPR increased significantly by 62.96% relative to SSR, whereas Blastocladiomycota under NTR was significantly higher than in the other treatments, with increases of 4.49–31.40-fold (*p* < 0.05). Interannual comparisons indicated that pronounced temporal turnover occurred mainly over 2020–2024: Ascomycota decreased from 2020 to 2022–2024 in CK and NTR (CK: 31.56–32.29% decrease; NTR: 34.48% decrease), and also decreased from 2020 to 2024 in SSR (43.56% decrease). In contrast, Basidiomycota increased from 2020 to 2024 in CK and SSR, with increases of 225.94 and 228.14%, respectively (*p* < 0.05). In addition, in DPR, Mortierellomycota increased from 2020 to 2022 (305.79% increase) and Chytridiomycota increased from 2020 to 2021 (962.44% increase). Glomeromycota increased from 2020 to 2022 in both SSR and NTR, with increases of 1120.41 and 1236.36%, respectively (*p* < 0.05).

At the genus level, in 2024, *Solicoccozyma* in SSR and NTR increased significantly by 2.94-fold and 3.00-fold, respectively, compared with DPR (*p* < 0.05; Figure 3B). *Trichosporiella* in DPR was significantly higher than in CK, SSR, and NTR, with increases of 14.18-fold, 5.58-fold, and 29.74-fold, respectively (*p* < 0.05). Across years, *Fusarium* showed significant fluctuations in CK: in 2020 it was significantly higher than in 2021 and 2024 by 2.68-fold and 2.48-fold, respectively, and in 2023 it was significantly higher than in 2021 and 2024 by 2.09-fold and 1.93-fold, respectively (*p* < 0.05). *Pyrenochaetopsis* exhibited significant interannual variation only in NTR, with 2022 and 2023 being significantly higher than 2024 by 68.23 and 68.31%, respectively (*p* < 0.05). Meanwhile, interannual variation in *Trichosporiella* was more pronounced across treatments: in CK, 2021 was significantly higher than 2022, 2023, and 2024 by 2.09-fold, 2.68-fold, and 1.77-fold, respectively; in DPR, 2023 and 2024 were significantly higher than 2021 by 5.88-fold and 6.89-fold, respectively; and in NTR, 2021 was significantly higher than 2020 by 5.35-fold (*p* < 0.05). Multiple-comparison results further indicated clear treatment effects: *Fusarium* in CK increased by 84.16% relative to DPR and by 1.04-fold relative to SSR (*p* < 0.05); *Pyrenochaetopsis* in CK increased by 64.30% relative to DPR (*p* < 0.05). *Trichosporiella* was significantly higher in DPR than in CK, SSR, and NTR, increasing by 5.84-fold versus CK, 2.53-fold versus SSR, and 15.31-fold versus NTR (*p* < 0.05), indicating a stronger enrichment effect of DPR on this genus and suggesting that it may play a key role in driving compositional differentiation among treatments.

### 3.4 Functional trait profiles of soil fungal communities under different straw-returning practices

**Figure 4:**
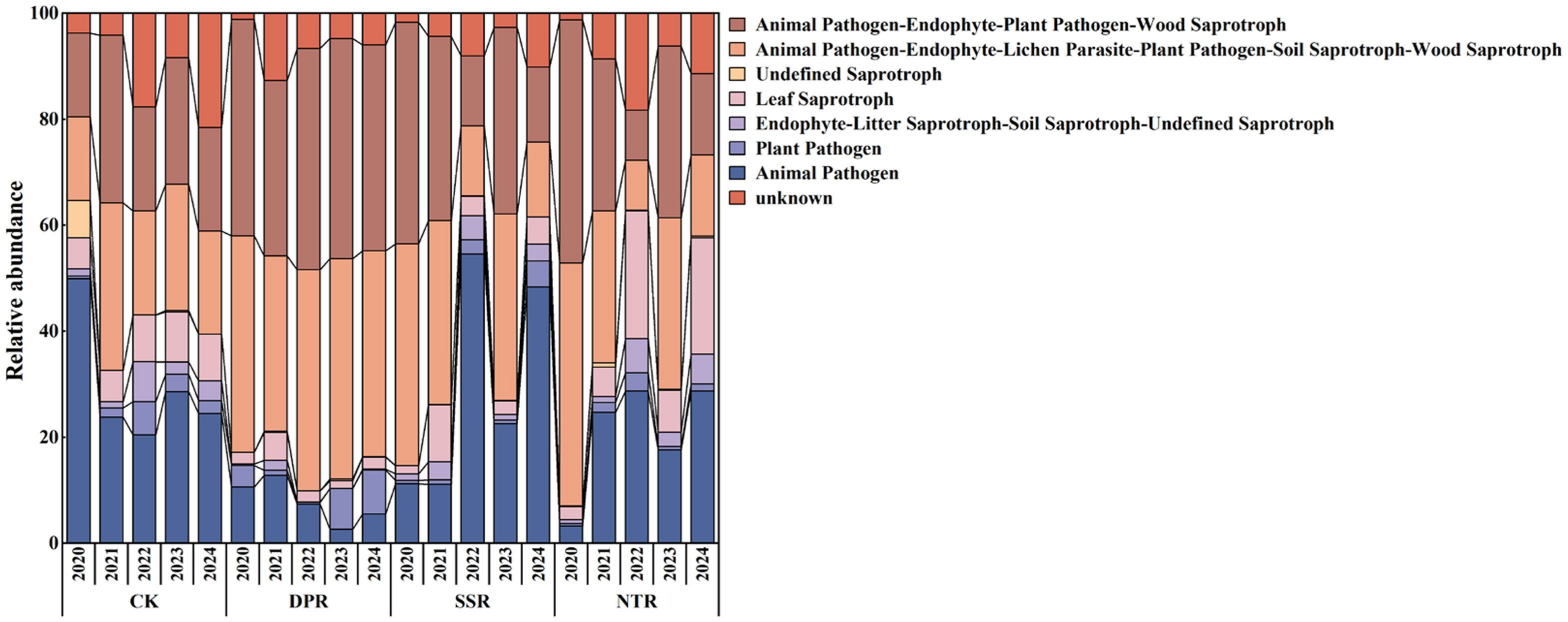
Functional guild distribution of soil fungal communities under different straw-returning practices from 2020 to 2024.

Functional guild inference indicated that different straw-returning practices significantly altered the functional structure of soil fungal communities, with the main differences reflected in the relative abundances of three guild categories: Pathogen, Saprotroph, and symbiotic fungi (Figure 4). In the among-treatment comparison for 2024, Pathogen differed significantly only between SSR and DPR, with SSR being 1.63-fold higher than DPR (*p* < 0.05). Symbiotic fungi also showed a significant difference only between DPR and SSR, with DPR being 2.31-fold higher than SSR (*p* < 0.05), whereas Saprotroph did not differ significantly among treatments (*p* > 0.05). However, it should be noted that FUN Guild-based functional assignments are inference-based and may not fully reflect the actual functional activities of fungal communities.

Across years, Pathogen showed no significant interannual variation within any treatment (CK, DPR, SSR, or NTR; *p* > 0.05). Saprotroph exhibited significant interannual variation only in SSR, where values in 2021 were 5.51-, 3.03-, 3.05-, and 1.93-fold those in 2020, 2022, 2023, and 2024, respectively (*p* < 0.05). Symbiotic fungi showed no significant interannual variation in CK or DPR (*p* > 0.05) but fluctuated significantly in SSR and NTR. In SSR, symbiotic fungi in 2020 were 2.69- and 2.74-fold higher than in 2022 and 2024, respectively, and 26.45% higher than in 2023 (*p* < 0.05). In NTR, symbiotic fungi in 2020 were 1.14- and 1.99-fold higher than in 2021 and 2024, respectively, and values in 2023 were 1.90-fold higher than in 2022 (*p* < 0.05).

Overall treatment-effect comparisons further showed that Pathogen was significantly higher in CK and SSR than in DPR (both 1.15-fold higher than DPR; *p* < 0.05). Saprotroph was significantly higher in NTR than in DPR and SSR (2.63-fold vs. DPR and 1.32-fold vs. SSR), and CK was also significantly higher than DPR (1.64-fold; *p* < 0.05). Symbiotic fungi were significantly higher in DPR than in CK, SSR, and NTR, increasing by 90.81, 35.50, and 51.22%, respectively; additionally, SSR was significantly higher than CK by 40.82% (*p* < 0.05).

### 3.5 Effects of straw-returning practices on soil fungal community assembly

**Figure 5:**
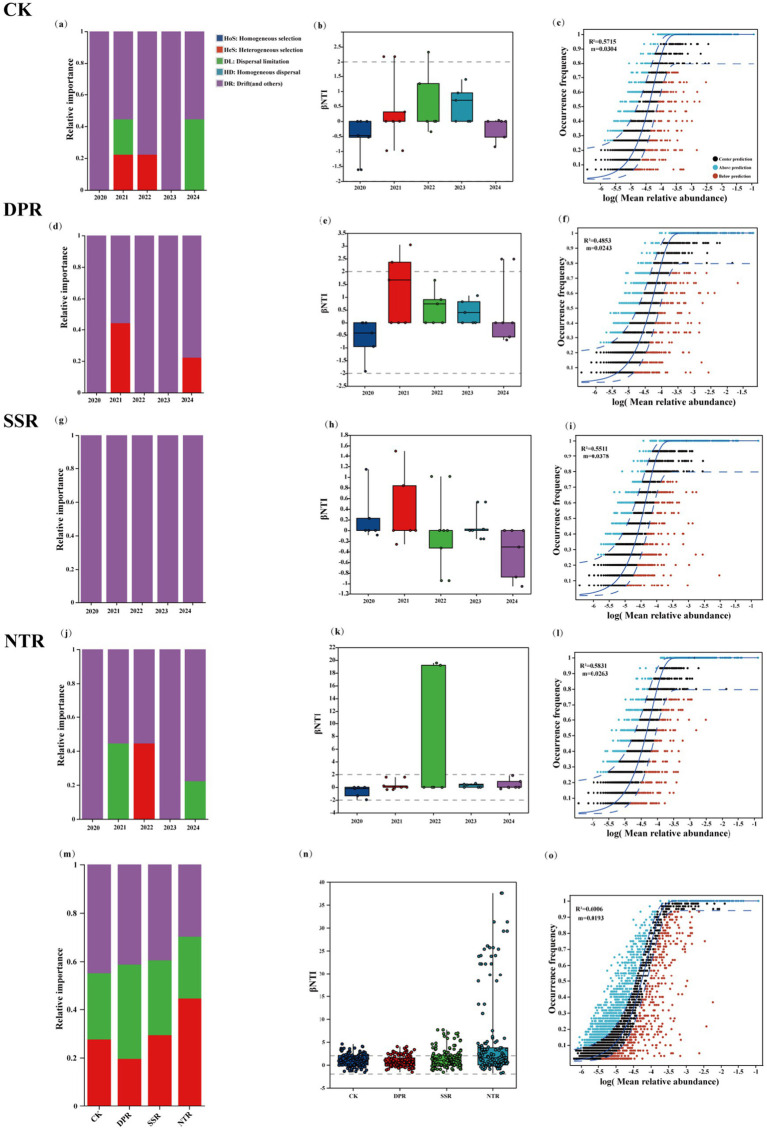
Assembly processes, *β*NTI distributions, and neutral community model (NCM) fits of soil fungal communities under different straw-returning practices. **(a–c)** CK; **(d–f)** DPR; **(g–i)** SSR; **(j–l)** NTR; **(m–o)** overall comparisons. **(a,d,g,j)** Show interannual changes (2020–2024) in the relative contributions of assembly processes inferred from null-model partitioning. **(b,e,h,k)** Present interannual βNTI distributions for each treatment (dashed lines indicate the βNTI = ±2 thresholds). **(c,f,i,l)** Show treatment-specific NCM fits; solid lines represent the best fit and dashed lines represent the 95% confidence intervals of model predictions, with point colors indicating OTU occurrence frequencies deviating above or below the prediction. **(m)** Compares the relative contributions of assembly processes among treatments; **(n)** compares βNTI distributions among treatments; and **(o)** shows the overall NCM fit (pooled samples). Parameters *m* and R^2^ denote the estimated immigration rate and the goodness of fit, respectively. Error bars represent standard deviation (SD) based on three replicates. For a given treatment, different lowercase letters indicate significant differences among years (*p* < 0.05), whereas within a given year, different uppercase letters indicate significant differences among treatments (*p* < 0.05).

Different straw-returning practices significantly altered the composition of soil fungal community assembly processes and their interannual dynamics. Assembly-process attribution indicated that SSR maintained a relatively stable process structure throughout the study period, whereas DPR and NTR exhibited signals of heterogeneous selection in 2022; consistently, βNTI results showed a significant increase in βNTI for NTR in 2022 (Figure 5j). In 2024, null-model results further indicated that assembly was dominated by stochastic processes across treatments: the undominated fraction accounted for 55.56, 77.78, and 55.56% in CK, DPR, and NTR, respectively, and 100% in SSR; dispersal limitation was detected only in CK (44.44%) and NTR (22.22%); heterogeneous selection accounted for 22.22% in both DPR and NTR, whereas homogeneous selection and homogenizing dispersal were 0 in all treatments (Figures 5a,d,g,j). Assembly stability analysis showed that DPR exhibited the smallest interannual fluctuations, whereas NTR showed higher variability. Neutral community model fitting suggested that, in 2024, the overall goodness of fit was R^2^ = 0.5132 with an estimated immigration rate of m = 0.0314 (Figure 5); treatment-specific fits were R^2^ = 0.5715, 0.5511, and 0.5831 for CK, SSR, and NTR, respectively, and R^2^ = 0.4853 for DPR (Figures 5c,f,i,l). Notably, a higher proportion of OTUs in NTR deviated from the model-predicted confidence interval (Figure 5l). Collectively, these results indicate that straw-returning practices differ in the relative contribution of stochastic processes, the timing of selection signals, and assembly stability, thereby leading to divergent fungal assembly patterns among treatments (Figure 5).

### 3.6 Co-occurrence network analysis of soil fungal communities under different straw-returning practices

**Figure 6:**
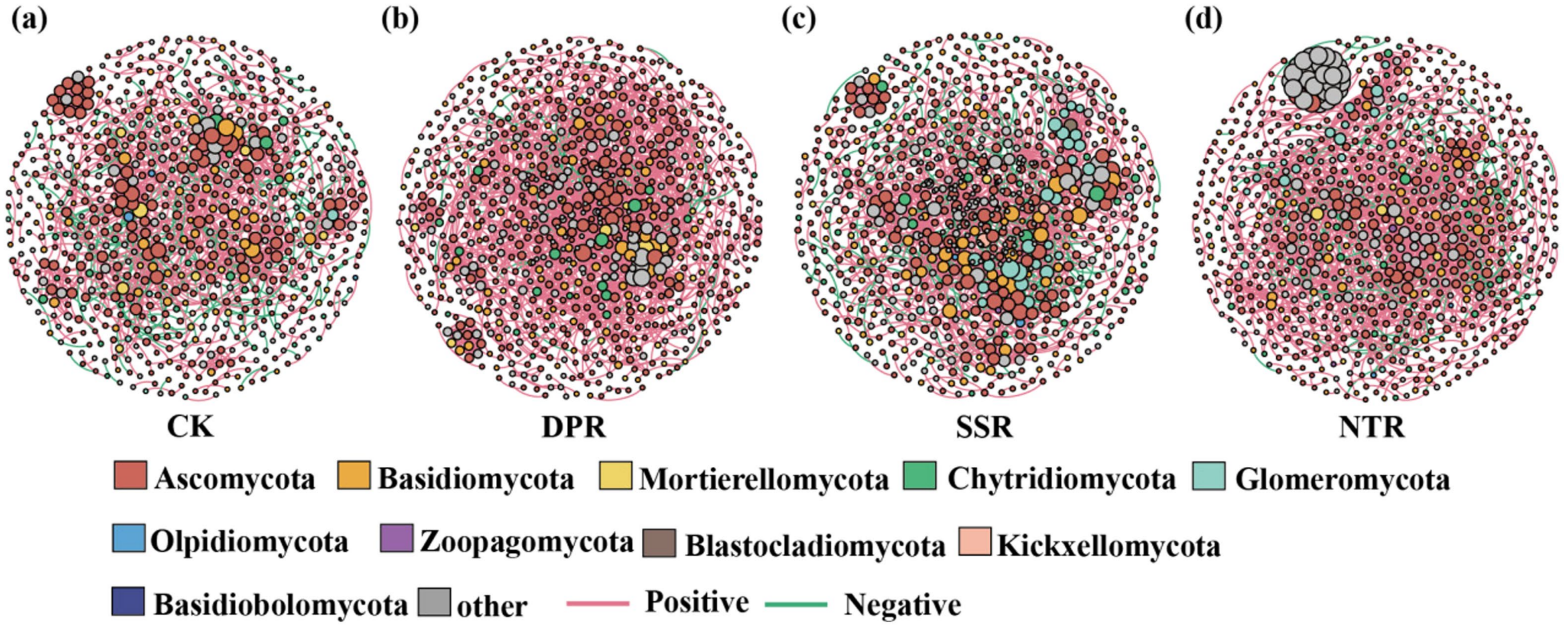
Soil fungal co-occurrence networks under different straw-returning practices: **(a)** CK, **(b)** DPR, **(c)** SSR, and **(d)** NTR. Node size is proportional to node degree, and node color indicates fungal phyla. Edges represent correlations between nodes (red, positive; green, negative).

Different straw-returning practices significantly affected the topological properties of soil fungal co-occurrence networks (Figures 6a–d). The NTR network had the largest size, comprising 774 nodes and 2,361 edges, with a mean degree of 6.101 and a graph density of 0.008, indicating higher network complexity and connectivity. The DPR network exhibited a relatively high average clustering coefficient (0.405) and a high proportion of positive edges (93.61%), suggesting a greater prevalence of potential cooperative associations. The SSR network showed the highest clustering coefficient (0.410) among the four treatments, but also had the highest proportion of negative edges (18.98%), implying relatively stronger potential antagonistic/competitive associations. The CK network had the highest modularity (0.829), and also the largest average path length (6.979) and network diameter (24), indicating weaker overall connectivity and more pronounced modular differentiation (Table 2).

**Table 2:** Summary of topological properties of soil fungal co-occurrence networks under different straw-returning practices.

Topological features	CK	DPR	SSR	NTR
Nodes	698	767	689	774
Edges	1,419	2017	1,216	2,361
Average degree	4.066	5.035	4.155	6.101
Network diameter	24	16	17	18
Graph density	0.006	0.006	0.005	0.008
Modularity	0.829	0.76	0.8	0.7664
Average clustering coefficient	0.359	0.405	0.41	0.385
Average path length	6.979	5.991	6.087	5.699
Positive edges (%)	0.8266	0.9361	0.8102	0.8806
Negative edges (%)	0.1734	0.0639	0.1898	0.1194

**Figure 7:**
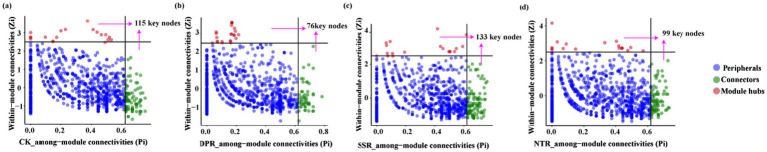
Zi–Pi plots derived from soil fungal co-occurrence network topology, showing the distribution of OTU node roles under different straw-returning practices: **(a)** CK, **(b)** DPR, **(c)** SSR, and **(d)** NTR.

Zi–Pi analysis indicated that the distribution of key nodes in soil fungal co-occurrence networks differed among straw-returning practices (Figures 7a–d). SSR identified the greatest number of key nodes (*n* = 133), followed by CK (*n* = 115) and NTR (*n* = 99), whereas DPR had the fewest (*n* = 76). Across treatments, key nodes were predominantly affiliated with Ascomycota, and their relative abundance was generally higher under straw-return treatments than under CK. No network hubs were detected in any treatment; key nodes were mainly classified as module hubs and connectors. Overall, straw-returning practices reshaped the network positions and functional roles of key fungal taxa, reflecting treatment-specific differences in the organization of soil microbial communities and their potential ecological functions.

### 3.7 Relationships between soil fungal community dynamics and environmental factors

**Figure 8:**
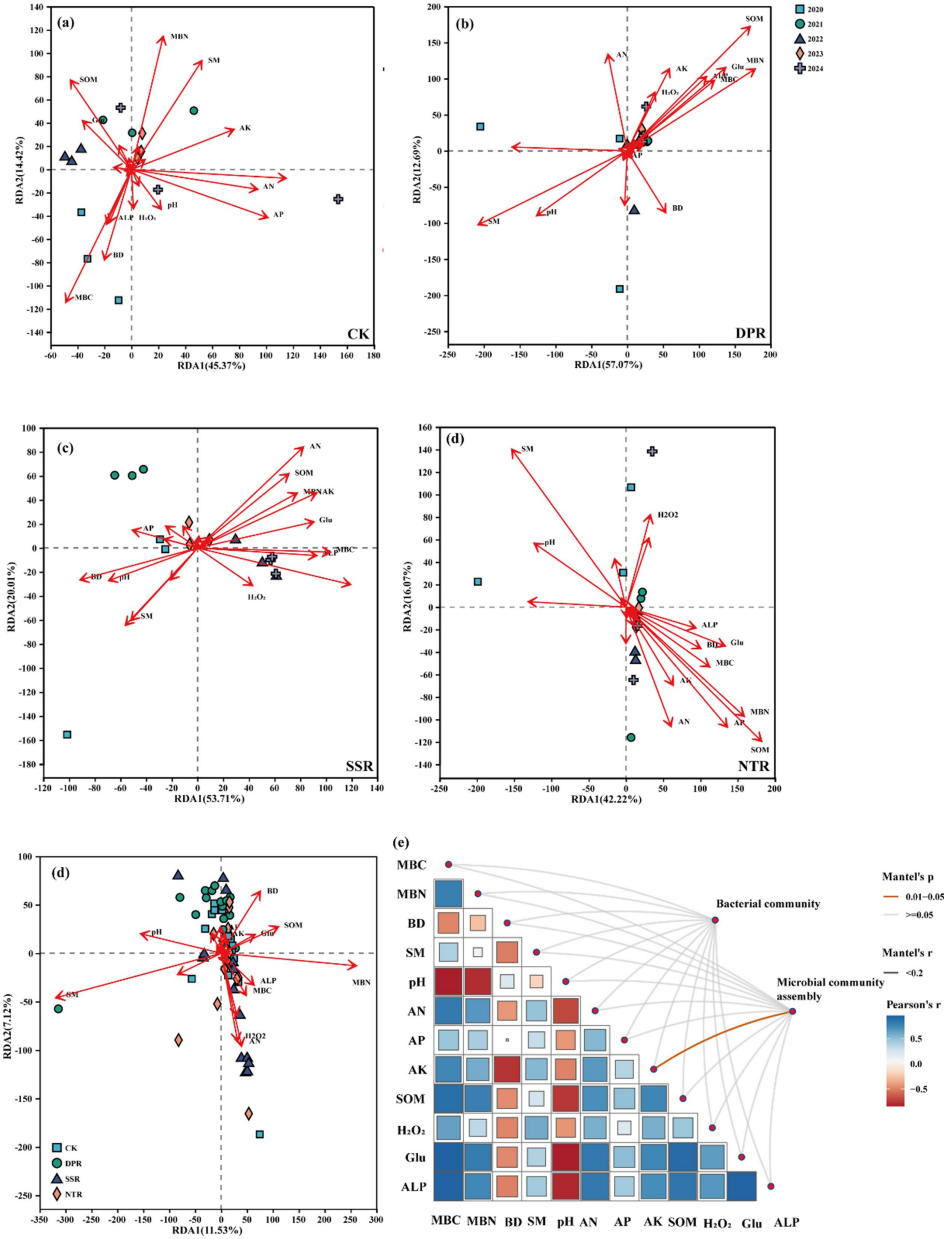
Redundancy analysis (RDA) showing relationships between soil fungal communities and soil properties under different straw-returning practices **(a–d)**, and the key environmental factors driving fungal community composition and assembly processes **(e)**.

RDA identified the key environmental drivers associated with differences in soil fungal community structure under contrasting straw-returning practices (Figure 8e). Across 2020–2024, MBN (*p* = 0.003), SM (*p* = 0.003), MBC (*p* = 0.002), and SOM (*p* = 0.005) were significantly associated with soil fungal community structure. Notably, MBN (*p* = 0.002) emerged as an important factor shaping fungal community structure (Figures 8a–d). To further disentangle the pathways linking soil nutrients, physicochemical properties, microbial biomass, fungal functional guilds, community diversity, and network complexity under different straw-returning practices, we constructed a partial least squares path model (PLS-PM). In addition, Mantel tests indicated that soil physicochemical properties and microbial attributes were significantly correlated with fungal community composition and assembly processes in CK, DPR, SSR, and NTR (Figure 8). Moreover, fungal life-history strategy–related metrics were positively correlated with available potassium (AK) (Figure 8f, *p* < 0.05).

**Figure 9:**
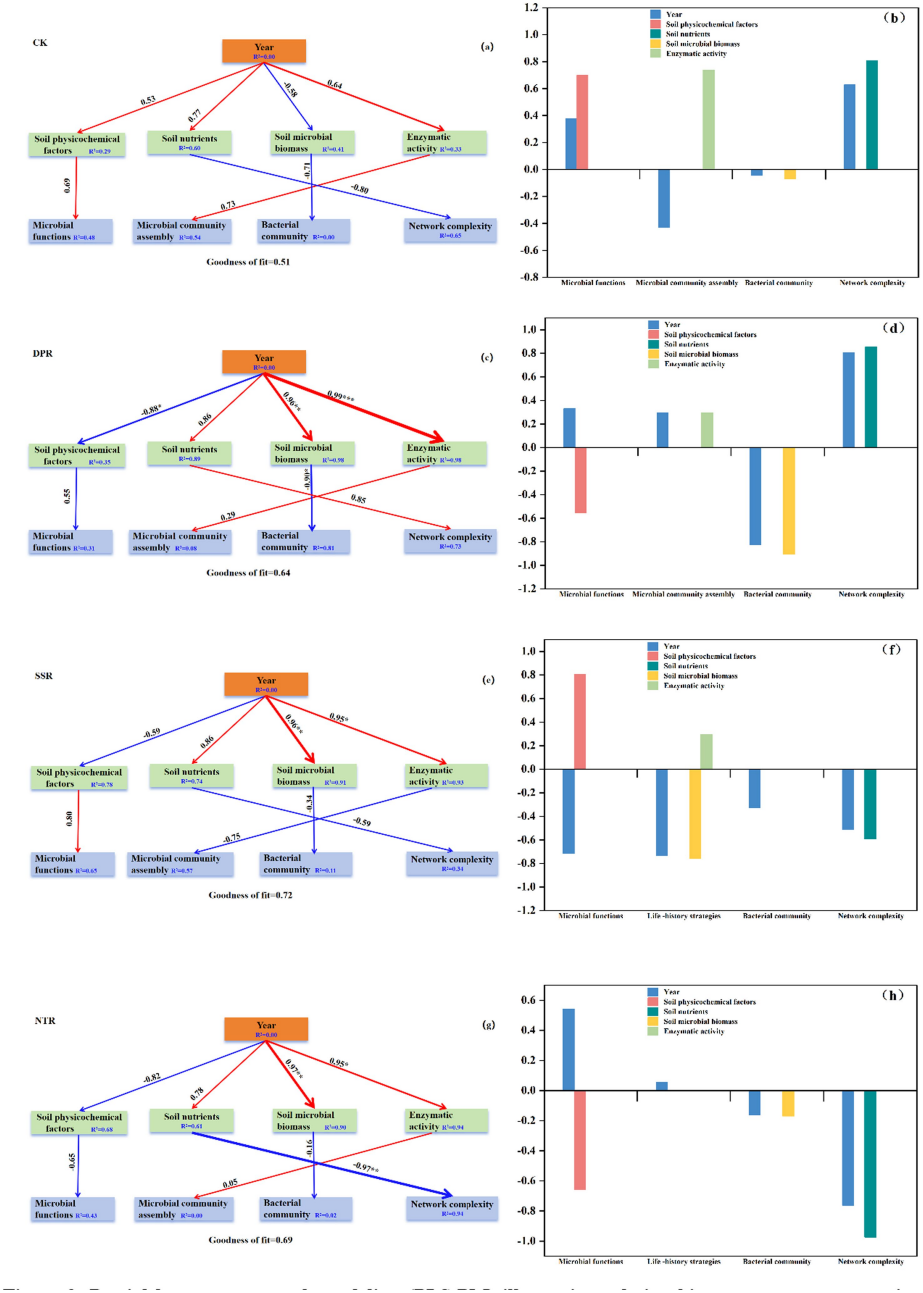
Partial least squares path modeling (PLS-PM) illustrating relationships among straw-returning practices, soil properties, fungal community diversity, functional guild structure, network complexity, and community assembly under different treatments: **(a, b)** CK, **(c, d)** DPR, **(e, f)** SSR, and **(g, h)** NTR. Panels **a, c, e**, and **g** show the PLS-PM results, and panels **b, d, f**, and **h** show the standardized total effects of year, soil physicochemical factors, soil nutrients, soil microbial biomass, and enzymatic activity on microbial functions, microbial community assembly/life-history strategies, bacterial community, and network complexity. Red and blue arrows indicate positive and negative path coefficients, respectively. *p < 0.05, **p < 0.01, ***p < 0.001.

Based on the PLS-PM results (Figure 9), the pathways linking key soil factors to fungal ecological processes differed markedly among straw-returning practices. Overall, year acted as a core exogenous driver and was indirectly associated with functional guild structure, community diversity, and network complexity primarily through intermediate variables, including soil physicochemical properties, nutrient status, microbial biomass, and enzyme activities. In CK, soil nutrients and microbial biomass showed negative path coefficients with fungal diversity and network complexity (−0.80 and −0.71), whereas physicochemical properties and enzyme activities exerted positive effects on functional guild structure (0.69 and 0.73) (Figure 9a). In DPR, microbial biomass had a significant negative effect on fungal diversity (−0.90*), and physicochemical properties were also negatively associated with functional guild structure (−0.55); in contrast, enzyme activities and nutrient status positively influenced community assembly and network complexity (0.29 and 0.85, respectively) (Figure 9b). In SSR, soil nutrients and microbial biomass negatively affected community assembly and network complexity, whereas year consistently exerted positive effects on multiple variables. This model showed the highest goodness of fit (GoF = 0.72), indicating comparatively clearer pathways (Figure 9c). In NTR, soil nutrients had a significant direct negative effect on fungal network complexity (−0.97**), suggesting that higher nutrient availability may suppress the development of network complexity (Figure 9d). Standardized total-effect analysis further indicated that year, enzyme activities, and microbial biomass contributed most to functional guild structure, community assembly, and diversity across treatments, highlighting the coupled effects of straw-return duration and key soil factors in shaping microbial ecological processes.

## 4 Discussion

### 4.1 Effects of consecutive-year straw-returning practices on soil fungal diversity

Soil fungi are key indicators of soil quality and ecological functioning and are crucial for the sustainability of agroecosystems (Raza et al., 2023). Long-term straw return significantly altered the ecological characteristics of soil fungal communities, including diversity, community composition, assembly mechanisms, and the succession of functional guilds (Wang et al., 2024b). This study showed that the DPR treatment significantly increased soil fungal *α*-diversity across years, particularly in the Shannon and ACE indices, with values significantly higher than those under the other treatments (Figure 1). This pattern may be attributable to DPR-driven straw comminution and the associated improvement in soil physicochemical conditions (e.g., pH and SOM), which likely provides fungi with more stable and heterogeneous niches (Wang et al., 2025). In contrast, although NTR increased diversity in some years, it exhibited larger interannual fluctuations, suggesting that its effects on fungal communities are more contingent on climatic variability and changes in soil condition (Louw et al., 2022). Ma et al. (2024) reported that DPR enhanced fungal diversity by improving soil physical properties and increasing soil organic matter, thereby creating a more favorable microbial habitat and significantly increasing both species richness and diversity. In comparison, the diversity-promoting effect of NTR was weaker than that of DPR. Although NTR increased species richness to some extent, improvements in overall diversity and community stability were relatively slow, likely because its benefits were mediated primarily through gradual improvements in soil structure and moisture conditions rather than direct stimulation of community richness (Dai et al., 2024). These results confirm that straw return plays an important role in shaping soil fungal diversity and further indicate that different straw-returning practices differentially influence microbial diversity by regulating soil physicochemical properties (Zhang et al., 2023). The effects of straw return depend not only on the method of residue incorporation but also on associated changes in soil structure, substrate availability for microbes, and habitat space (Liu et al., 2023). This study demonstrated the integrated effects of soil physicochemical properties, management practices, and soil structural dynamics on microbial community diversity under different straw-returning regimes (Figure 2). These findings advance our understanding of the dynamic responses of microbial diversity in agroecosystems and provide both a theoretical basis and practical guidance for developing straw-returning strategies with greater ecological benefits.

### 4.2 Effects of consecutive-year straw return under different practices on soil fungal community structure and function

Different straw-returning practices significantly modulated fungal phylum-level composition and genus-level abundances, with pronounced temporal dynamics across years (Figure 3). The DPR treatment likely promoted the proliferation of symbiotic fungi (e.g., Trichosporiella) by improving soil physical conditions, enhancing the distribution of soil organic matter, and increasing microbial substrate availability (Whalen et al., 2024). In particular, the enrichment of *Trichosporiella* may be linked to its functional role in organic matter decomposition and carbon cycling, potentially enhancing carbon turnover and nutrient release, thereby contributing to improved soil fertility and sustainable cropland management. This treatment not only improved the microbial habitat but also likely enhanced the establishment of beneficial taxa, thereby supporting soil ecological functioning. Moreover, DPR effectively reduced the relative abundances of pathogenic fungi (e.g., Fusarium and Pyrenochaetopsis), highlighting its potential to lower pathogen load and improve soil health (Wang S. et al., 2025). This pattern suggests that moderate tillage disturbance coupled with organic carbon inputs can optimize soil microbial community structure and enhance diversity and stability, thereby playing a key role in promoting crop growth and improving soil health (Wang Y. et al., 2017). In contrast, no-tillage straw mulching (NTR) increased the abundance of saprotrophic fungi (e.g., members of Chytridiomycota), but exerted weaker suppression of pathogens and showed larger interannual fluctuations in symbiotic taxa. This indicates that the regulatory effects of NTR on soil microbial communities may be less stable, potentially due to reduced topsoil disturbance that promotes heterogeneous substrate distribution and increased habitat heterogeneity (Pajares-Murgó et al., 2025). The SSR treatment likely promoted the growth of Basidiomycota by improving soil structure and water-holding capacity; these fungi play important roles in organic matter decomposition and soil nutrient cycling (Zhang et al., 2020). However, SSR showed a weaker suppressive effect on pathogens (e.g., Fusarium) and did not effectively reduce their abundance, which may be related to its comparatively limited capacity to restructure the microbial community (Trivedi et al., 2017).

Functional guild annotation corroborated the above compositional shifts and their associated functional responses (Figure 4). Under DPR, the relative abundance of symbiotic fungi increased significantly (by 35.50–90.81%), whereas pathogen-associated guilds showed a consistent declining trend, suggesting that DPR may facilitate the formation of microbial networks dominated by positive interactions and thereby support soil ecological functioning and crop health. This is consistent with the findings of Li Z. et al. (2023) and Li C. et al. (2023), who showed that improvements in soil structure and the activation of soil organic matter can effectively enhance the colonization potential and niche stability of symbiotic guilds.

### 4.3 Effects of consecutive-year straw return under different practices on soil fungal co-occurrence networks and community assembly

Microbial turnover is a key process governing ecosystem functioning, and the assembly of root-associated microbiomes—particularly those shaped by plant–microbe interactions—has received increasing attention (Li Z. et al., 2023; Li C. et al., 2023). Null-model analyses (Figure 5) indicated that fungal community assembly was dominated by stochastic processes, suggesting that community composition was primarily governed by ecological drift and dispersal limitation. Previous studies have shown that the contribution of stochasticity tends to increase with nutrient enrichment, whereas deterministic processes are more often associated with nutrient-poor condition (Xie et al., 2022). Accordingly, under NTR and SSR, soil fungal community assembly was primarily governed by stochastic processes, highlighting the important role of ecological drift in alleviating resource constraints and facilitating microbial diversity and dispersal. Enhanced stochastic processes may promote the immigration of taxa from the regional species pool into local communities, thereby helping to maintain ecological stability and functional heterogeneity, particularly under low-disturbance conditions where soil organic matter gradually accumulates (Gong et al., 2023). This pattern was further supported by the neutral community model, under which the fungal community in SSR showed a relatively good fit to neutral expectations (R^2^ > 0.5). Under such conditions, taxa may persist by balancing weak environmental filtering with resource limitation, thereby adapting to prevailing environmental constraint (Dang et al., 2024). Consequently, NTR and SSR exhibited consistently high stochasticity indices (>90%) across years, suggesting that straw return reduced the strength of environmental filtering and increased the relative importance of neutral dispersal as a dominant driver of community assembly (Wang et al., 2025). In contrast, in CK, selective processes such as environmental filtering played a more prominent role in community assembly, indicating that under conventional management, fungal assembly relies more strongly on constraints imposed by soil physicochemical conditions (Wang and Zou, 2020).

This study demonstrated that straw-return management markedly reshaped the structure of soil fungal co-occurrence networks, indicating that residue management can influence potential microbial associations and, consequently, soil ecosystem stability. The no-tillage straw mulching treatment (NTR) produced the largest network, with the greatest numbers of nodes and edges and a relatively high connectivity and mean degree (6.101), suggesting a higher prevalence of potential cooperative associations among fungal taxa. Such increased network complexity may enhance system resilience to environmental disturbances and support a broader range of ecological functions (Dai et al., 2023). Although the DPR network was smaller in size, it exhibited a high clustering coefficient (0.405) and the highest proportion of positive edges (93.61%), indicating tighter potential cooperative interactions and functional complementarity among microbial taxa. This suggests that moderate soil disturbance combined with organic carbon inputs may strengthen microbial coordination within the network (Liu et al., 2024). In contrast, although subsoiling straw incorporation (SSR) increased network modularity and clustering, it also showed a higher proportion of negative edges (18.98%), suggesting stronger potential antagonistic or competitive associations among microbial taxa. Such shifts may increase the risk of functional trade-offs or reduced system stability (Brinkmann et al., 2019). In the control (CK), where no organic carbon was returned, the network was relatively sparse, with the largest average path length and diameter and clearly delineated modules, indicating weak connectivity among microbial taxa and lower ecological stability (Hao et al., 2024).

Network topological analysis further revealed treatment-specific differences in the ecological roles of fungi. Ascomycota consistently emerged as a core group across all treatments, likely contributing broadly to organic matter decomposition, nutrient cycling, and plant symbioses (Maestre et al., 2015). SSR identified the largest number of module hubs and connectors (133 nodes), whereas DPR had fewer key taxa (76 nodes). Nevertheless, the DPR network showed stronger integration and functional coordination, suggesting a potential advantage in regulating ecosystem functions (Zhang et al., 2024). Moreover, we found that the network positions and roles of keystone taxa reflect the mechanisms through which they shape microbial community organization and associated ecological function (Manici et al., 2024). These network metrics and functional-role differences highlight potential pathways through which straw-returning practices may regulate soil health and support sustainable agricultural management.

### 4.4 Relationships between soil fungal community dynamics and environmental factors under consecutive-year straw-returning practices

Building on RDA and partial least squares path modeling (PLS-PM), this study elucidated the coupled mechanisms linking soil physicochemical factors to fungal ecological processes under different straw-returning practices. MBN (microbial biomass N), SM (soil moisture), MBC (microbial biomass C), and SOM (soil organic matter) emerged as key variables significantly associated with fungal community structure, with MBN showing the strongest signal (*p* = 0.002), underscoring the importance of microbial nutrient availability in regulating community organization (Li C. et al., 2023).

PLS-PM further indicated that year indirectly affected fungal diversity, functional guild structure, and network complexity via intermediate variables, including soil physicochemical properties, microbial biomass, and enzyme activities. Under DPR, enzyme activities and nutrient status were positively associated with network complexity (0.85 and 0.29, respectively), whereas microbial biomass and physicochemical factors showed negative effects on community assembly, suggesting that DPR may promote the expansion of niches for beneficial taxa by optimizing carbon inputs and substrate availability (Wu et al., 2023). In contrast, SSR and CK exhibited lower microbial network complexity, with negative pathways predominating, suggesting that ecological processes may be constrained in the absence of moderate disturbance and/or sufficient organic carbon inputs (Wallwork et al., 2022). In NTR, soil nutrients had a significant negative effect on network complexity (−0.97**), suggesting that no-tillage management may promote nutrient accumulation and thereby strengthen heterogeneous selection within microbial communities. Standardized total-effect analysis further confirmed the dominant roles of soil enzyme activities and microbial biomass in regulating microbial ecological functions (López-Aizpún et al., 2018). Overall, our findings provide a mechanistic basis for optimizing straw-returning strategies and offer scientific guidance for sustainable agricultural management.

Notably, DPR and NTR produced distinct responses in fungal diversity, functional guild structure, and co-occurrence network architecture. DPR enhanced fungal diversity and increased the abundance of symbiotic guilds while supporting a more stable network, suggesting beneficial implications for soil health and crop performance. In contrast, NTR increased network complexity but was accompanied by higher pathogen abundance, which may pose potential ecological risks. Although straw return generally exerted positive effects on microbial communities, the ecological outcomes varied among practices, highlighting the need for integrated, multi-criteria evaluation in management decisions. Future work should further resolve the dynamic links between straw return and soil microbial communities, with particular emphasis on the trade-offs between pathogen suppression and community stability, to better inform sustainable agriculture and soil health management.

## 5 Conclusion

Based on continuous observations during years 3–7 of straw return, different straw-returning practices significantly reshaped the temporal successional trajectories and assembly pathways of soil fungal communities. Fungal *α*-diversity increased during the mid-term phase and then stabilized in the later phase. Community composition exhibited directional turnover with increasing years: Ascomycota declined over time in CK and NTR, whereas Basidiomycota increased over time in CK and SSR. Key taxa also differed among treatments: in year 7, Ascomycota was significantly higher in DPR than in SSR, and Blastocladiomycota was significantly enriched in NTR. At the genus level, DPR significantly enriched *Trichosporiella*, whereas *Solicoccozyma* was relatively more abundant in SSR and NTR. Functionally, DPR tended to increase symbiotic guilds and showed stronger network cooperativity and higher stability, whereas NTR formed a larger network in year 7 but was accompanied by an increase in pathogen-associated guilds. Mechanistically, heterogeneous selection signals emerged in year 5 for DPR and NTR, with βNTI in NTR increasing to 8.61; by year 7, stochastic processes dominated overall. The neutral community model showed a moderate fit (R^2^ = 0.5132) with an estimated immigration rate of m = 0.0314, while NTR exhibited a higher proportion of OTUs deviating from the neutral prediction interval. Overall, years 4–5 represent a critical window for community differentiation and successional transition; DPR appears more conducive to stable optimization, whereas NTR warrants enhanced risk monitoring. The PLS-PM results further provide a mechanistic basis for designing management strategies to mitigate climate-induced soil degradation in semi-arid regions, particularly through optimizing straw-returning practices and regulating key soil physicochemical drivers.

## Data Availability

The data presented in this study are publicly available. The data can be found here: https://www.ncbi.nlm.nih.gov, accession PRJNA140523.

## Footnotes

1
http://rdp.cme.msu.edu/
