# Supplementary material for: From the 3rd to the 7th year after straw return, different straw-returning practices drive shifts in soil fungal community composition, functional differentiation, and the reconfiguration of community assembly processes
Source: Front Microbiol. 2026 Apr 21;17:1808010. doi: 10.3389/fmicb.2026.1808010 (PMC13141306; doi:10.3389/fmicb.2026.1808010)
Supplement: Supplementary file 1 [file Supplementary_file_1.docx]

**Supplementary Information**

**Table S1** The basic soil properties of the site sampled at 0–45 cm depth in 2020

| **Treatment** | **Year** | **Available nitrogen**  **(mg/kg)** | **Available phosphorus**  **(mg/kg)** | **Available potassium**  **(mg/kg)** | **Organic carbon**  **(g/kg)** |
| --- | --- | --- | --- | --- | --- |
| Farmers' shallow rotation （CK） | 2020 | 53.43 | 2.63 | 64.78 | 16.98 |
| Straw incorporated  with deep tillage  (DPR) | 2020 | 60.39 | 3.04 | 78.25 | 18.75 |
| straw incorporated with subsoiling  (SSR) | 2020 | 65.65 | 3.42 | 97.77 | 21.12 |
| no-tillage mulching straw return  (NTR) | 2020 | 72.84 | 3.75 | 113.17 | 22.18 |

**Table S****2** Comparison of physicochemical properties among different treatments

| **Treatment** | **Year** | **BD**  **(g /cm^−3^)** | **SM**  **(%)** | **AN**  **(mg/kg)** | **AP**  **(mg/kg)** | **AK**  **(mg/kg)** | **SOM**  **(g/kg)** |
| --- | --- | --- | --- | --- | --- | --- | --- |
| CK | 2020 | 1.60±0.01a | 15.91±1.10b | 53.43±4.11b | 2.63±0.06a | 64.78±2.56b | 16.98±0.46b |
|  | 2021 | 1.56±0c | 17.6±0.17ab | 53.07±0.75b | 2.56±0.04a | 66.06±0.26b | 18.81±0.18a |
|  | 2022 | 1.59±0.01ab | 17.66±0.19a | 53.48±0.2b | 2.53±0.04a | 65.58±0.77b | 18.81±0.18a |
|  | 2023 | 1.56±0bc | 18.46±0.23a | 55.26±1.32ab | 2.62±0.05a | 82.18±1.14a | 18.22±0.34a |
|  | 2024 | 1.59±0ab | 18.37±0.03a | 61.42±0.81a | 3.39±0.93a | 77.2±1.74a | 16.27±0.43b |
| DPR | 2020 | 1.45±0.02c | 31.3±1a | 72.84±2.31c | 3.75±0.16c | 113.1±5.46a | 22.18±1.17b |
|  | 2021 | 1.51±0b | 19.15±0.34b | 70.34±0.19c | 3.83±0.02b | 117.02±0.2a | 26.55±0.22a |
|  | 2022 | 1.58±0.01a | 19.49±0.14b | 73.92±0.61c | 4.72±0.06a | 92.63±1.74b | 26.55±0.22a |
|  | 2023 | 1.45±0.01c | 19.94±0.27b | 73.09±1.37b | 4.06±0.02b | 119.28±2.39a | 26.71±0.11a |
|  | 2024 | 1.41±0c | 20.15±0.39b | 78.16±1.34a | 3.67±0.05bc | 125.23±1.16a | 27.98±0.34a |
| SSR | 2020 | 1.52±0.01a | 23.27±0.28a | 62.43±7.01b | 3.84±0.3a | 85.85±1.8c | 18.3±0.55b |
|  | 2021 | 1.49±0ab | 20.09±0.07b | 74.16±1.23a | 3.49±0.11a | 99.02±0.79b | 25.48±0.08a |
|  | 2022 | 1.48±0.01ab | 20.43±0.11b | 74.24±1.07a | 3.65±0.04a | 102.68±1.09ab | 25.48±0.08a |
|  | 2023 | 1.5±0.02a | 20.24±0.4b | 74.44±1.11a | 3.67±0.05a | 102.44±1.56b | 25.58±0.23a |
|  | 2024 | 1.45±0.01b | 20.41±0.73b | 76.93±1.27a | 2.87±0.04b | 107.09±1.3a | 26.1±0.27a |
| NTR | 2020 | 1.48±0.01b | 26.41±0.89a | 61.26±5.73b | 2.78±0.17b | 89.98±5.17 | 21.01±0.41b |
|  | 2021 | 1.55±0a | 19.73±0.13b | 73.19±0.3a | 4.67±0.02a | 91.08±0.93b | 25.4±0.24a |
|  | 2022 | 1.46±0.01b | 20.29±0.43b | 71.77±0.4a | 4.01±0.07b | 120.31±1.46a | 25.4±0.24a |
|  | 2023 | 1.59±0.02a | 19.96±0.14b | 73.96±0.91a | 4.7±0.05a | 92.79±0.71b | 25.46±0.05a |
|  | 2024 | 1.6±0.01a | 20.79±0.45b | 75.94±0.38a | 4.7±0.05a | 94.24±1.37b | 25.87±0.3a |

*Values among treatments at the same soil depth followed by different letters (a, b, c) differed significantly at *P* < 0.05 (HSD test).CK,Farmer rotary tillage;DPR,Straw incorporation with deep tillage;SSR,Straw incorporation with Subsoiling;NTR,Straw incorporation with no-tillage.BD:Bulk density; SM: soil water content; AN,:soil alkaline dissolved nitrogen; SOM: Soil organic matter; AP: quick-acting phosphorus; AK:quick-acting potassium.

**Table S3** Comparison of enzyme activities among different treatments

| **Treatment** | **Year** | **H_2_O_2(_u/g/h)** | **GS(u/g/h)** | **ALP(u/g/h)** |
| --- | --- | --- | --- | --- |
| CK | 2020 | 1.61±0.06b | 40.7±0.67a | 65.34±0.74a |
|  | 2021 | 1.36±0.05b | 42.21±1.54a | 64.99±2.69a |
|  | 2022 | 1.36±0.05b | 43.36±4.3a | 49.55±2.07b |
|  | 2023 | 1.79±0.03a | 42.03±0.76a | 47.94±2.13b |
|  | 2024 | 1.48±0.03ab | 39.98±0.73a | 48.75±0.91b |
| DPR | 2020 | 2.16±0.01b | 60.95±0.5d | 181.58±5.02d |
|  | 2021 | 1.58±0.07c | 69.68±1c | 238.43±3.87d |
|  | 2022 | 1.53±0.15c | 75.39±0.57bc | 372.3±15.57c |
|  | 2023 | 3.29±0.04a | 82.07±4.92ab | 468±12.94b |
|  | 2024 | 3.28±0.11a | 92.5±2.32a | 514.35±4.3a |
| SSR | 2020 | 2.4±0b | 59±0.45c | 225.38±4.83d |
|  | 2021 | 1.61±0.01c | 66.72±1.63b | 237.41±6.85d |
|  | 2022 | 1.7±0.07c | 69.3±1.32b | 322.23±4.58c |
|  | 2023 | 3.31±0.05a | 75.08±0.75a | 436.52±15.73b |
|  | 2024 | 3.37±0.01a | 78.67±1.23a | 503.51±9.49a |
| NTR | 2020 | 2.03±0.03b | 56.79±1.17d | 106.54±2.24d |
|  | 2021 | 1.43±0.09c | 62.34±1.18cd | 116.44±6.42d |
|  | 2022 | 1.88±0.03b | 66.46±4.5bc | 180.24±13.3c |
|  | 2023 | 2.65±0.09a | 72.3±1.55ab | 279.07±5.75b |
|  | 2024 | 2.75±0.08a | 75.03±0.94a | 312.24±10.25a |

*Values among treatments at the same soil depth followed by different letters (a, b, c) differed significantly at *P* < 0.05 (HSD test). CK,Farmer rotary tillage;DPR,Straw incorporation with deep tillage;SSR,Straw incorporation with Subsoiling;NTR,Straw incorporation with no-tillage.ALP:soil alkaline phosphatase ; H_2_O_2_:catalase ;GS:glutaminase synthetase .


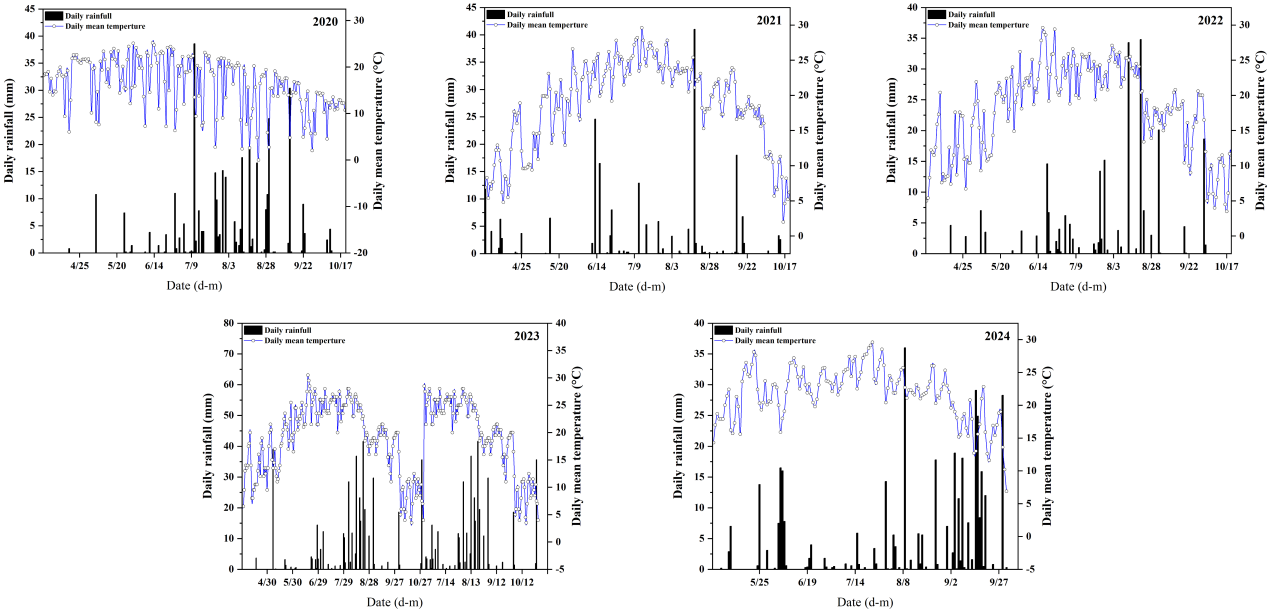


**Fig.S1** Main meteorological factors during the growing period in the experimental area
